# CT imaging-based approaches to cochlear duct length estimation—a human temporal bone study

**DOI:** 10.1007/s00330-021-08189-x

**Published:** 2021-08-31

**Authors:** Tabita Breitsprecher, Anandhan Dhanasingh, Marko Schulze, Markus Kipp, Rami Abu Dakah, Tobias Oberhoffner, Michael Dau, Bernhard Frerich, Marc-André Weber, Soenke Langner, Robert Mlynski, Nora M. Weiss

**Affiliations:** 1 Department of Otorhinolaryngology, Head and Neck Surgery „Otto Körner“, Rostock University Medical Center, Doberaner Strasse 137-139, D-18057 Rostock, Germany; 2 MED-EL, Fürstenweg 77a, A-6020 Innsbruck, Austria; 3 Rostock University Medical Center, Institute of Anatomy, Gertrudenstrasse 9, Rostock, Germany; 4 Rostock University, 18051 Rostock, Germany; 5 Department of Oral, Maxillofacial and Facial Plastic Surgery, Rostock University Medical Center, Schillingallee 35, D-18057 Rostock, Germany; 6 Department of Radiology, Rostock University Medical Center, Schillingallee 35, D-18057 Rostock, Germany

**Keywords:** Cochlea, Acoustic stimulation, Imaging, three-dimensional, Electrodes, implanted

## Abstract

**Objectives:**

Knowledge about cochlear duct length (CDL) may assist electrode choice in cochlear implantation (CI). However, no gold standard for clinical applicable estimation of CDL exists. The aim of this study is (1) to determine the most reliable radiological imaging method and imaging processing software for measuring CDL from clinical routine imaging and (2) to accurately predict the insertion depth of the CI electrode.

**Methods:**

Twenty human temporal bones were examined using different sectional imaging techniques (high-resolution computed tomography [HRCT] and cone beam computed tomography [CBCT]). CDL was measured using three methods: length estimation using (1) a dedicated preclinical 3D reconstruction software, (2) the established A-value method, and (3) a clinically approved otosurgical planning software. Temporal bones were implanted with a 31.5-mm CI electrode and measurements were compared to a reference based on the CI electrode insertion angle measured by radiographs in Stenvers projection (CDL_reference_).

**Results:**

A mean cochlear coverage of 74% (SD 7.4%) was found. The CDL_reference_ showed significant differences to each other method (*p* < 0.001). The strongest correlation to the CDL_reference_ was found for the otosurgical planning software-based method obtained from HRCT (CDL_SW-HRCT_; *r* = 0.87, *p* < 0.001) and from CBCT (CDL_SW-CBCT_; *r* = 0.76, *p* < 0.001). Overall, CDL was underestimated by each applied method. The inter-rater reliability was fair for the CDL estimation based on 3D reconstruction from CBCT (CDL_3D-CBCT_; intra-class correlation coefficient [ICC] = 0.43), good for CDL estimation based on 3D reconstruction from HRCT (CDL_3D-HRCT_; ICC = 0.71), poor for CDL estimation based on the A-value method from HRCT (CDL_A-HRCT_; ICC = 0.29), and excellent for CDL estimation based on the A-value method from CBCT (CDL_A-CBCT_; ICC = 0.87) as well as for the CDL_SW-HRCT_ (ICC = 0.94), CDL_SW-CBCT_ (ICC = 0.94) and CDL_reference_ (ICC = 0.87).

**Conclusions:**

All approaches would have led to an electrode choice of rather too short electrodes. Concerning treatment decisions based on CDL measurements, the otosurgical planning software-based method has to be recommended. The best inter-rater reliability was found for CDL_A-CBCT_, for CDL_SW-HRCT_, for CDL_SW-CBCT_, and for CDL_reference_.

**Key Points:**

• *Clinically applicable calculations using high-resolution CT and cone beam CT underestimate the cochlear size*.

• *Ten percent of cochlear duct length need to be added to current calculations in order to predict the postoperative CI electrode position*.

• *The clinically approved otosurgical planning software-based method software is the most suitable to estimate the cochlear duct length and shows an excellent inter-rater reliability*.

## Introduction

The spectral information of a cochlear implant (CI) electrode resembles Greenwood’s function, which matches the inner ear’s hair cells and corresponding spiral ganglion neurons to the stimulation frequency [1]. This knowledge may contribute to optimize fitting strategies and the representation of spectral information to the auditory pathway. Variations in size and shape of the cochlea can affect the CI electrode position as well as the cochlear coverage (CC) and consequently the final pitch discrimination [2–13]. In cases of surgery under the aim of residual hearing preservation, an individual choice of electrode length is favorable and several proposals to approach the appropriate electrode length depending on the cochlear duct length (CDL) are made [3, 14]. The best speech perception is reported in a CC between 70 and 75% [15]. This is in line with earlier studies that could not find a benefit from very deep insertion [16, 17]. A too short electrode insertion may lead to poorer functional outcomes [18]. When determining the CC for an optimal stimulation by a CI, it is important to understand the individual effects of the cochlear geometry on the choice of different electrode lengths. There are suggestions to adapt the electrode to the individual length of the cochlea under the aim of structure preservation for acoustic stimulation with hearing aids and optimized compensation of the profound hearing loss by electrical stimulation [19]. For this purpose, the dimensions of the cochlea may be measured using routine radiological imaging of the temporal bone. In advance to CI surgery, high-resolution computed tomography (HRCT) or cone beam computed tomography (CBCT) of the temporal bone is performed in clinical routine, offering the opportunity of analysis of the individual surgical site [20]. There are several approaches to estimate the CDL, mostly using volume-generated methods based on HRCT and magnetic resonance imaging (MRI) [21–24]. The CDL appears to be an important factor affecting CI outcomes and encouraging research to establish strategies for electrode selection [25]. However, it is unknown whether these methods are suitable for a reliable assessment of fine structured cochlear dimensions. In the experimental setting, synchrotron imaging is considered the most accurate imaging modality, but cannot be applied in clinical routine [26]. For CDL estimation from clinical imaging and selecting a suitable CI electrode, the spline curve method is assumed to lead to the most suitable results [23]. Nevertheless, reliable methods to perform CDL measurements are still demanded [24].

The goal of the present study is to determine (i) the most reliable radiological imaging and imaging software for measuring cochlear dimensions in clinical routine and (ii) which method is suitable to accurately predict the actual insertion depth of the CI electrode in order to provide patients with optimal conditions for combined cochlear stimulation with hearing aids and CI.

## Methods

The study protocol was approved by the local Ethics Committee in accordance with the Helsinki declaration (registration number: A2019-0089). Fresh temporal bones used in this study originated from the university donor program at the local Institute of Anatomy. All patients registered voluntarily to donate their body for medical scientific research and gave written informed consent during lifetime.

A total of 20 fresh human temporal bones were examined using two different cross-sectional imaging techniques used in clinical diagnostic workup before CI surgery, i.e., HRCT and CBCT. HRCT examinations were performed using a 64-row multidetector CT (Aquilion, Canon Medical Systems) with 0.5-mm collimation (120 kVP, 150 mAs) and a 512 × 512 matrix. Images were reconstructed using a bone kernel (slice thickness: 0.2 mm). CBCT examinations were performed using a Pax-Zenith 3D (VATECH Co. Ltd.; capture software: byzz, version 5.8.3, Orangedental). The temporal bone was positioned analogous to the patient’s upright position in CBCT. The field of view (FOV) was set to 9 × 12 cm; the voxel size was 0.2 mm.

**Fig. 1:**
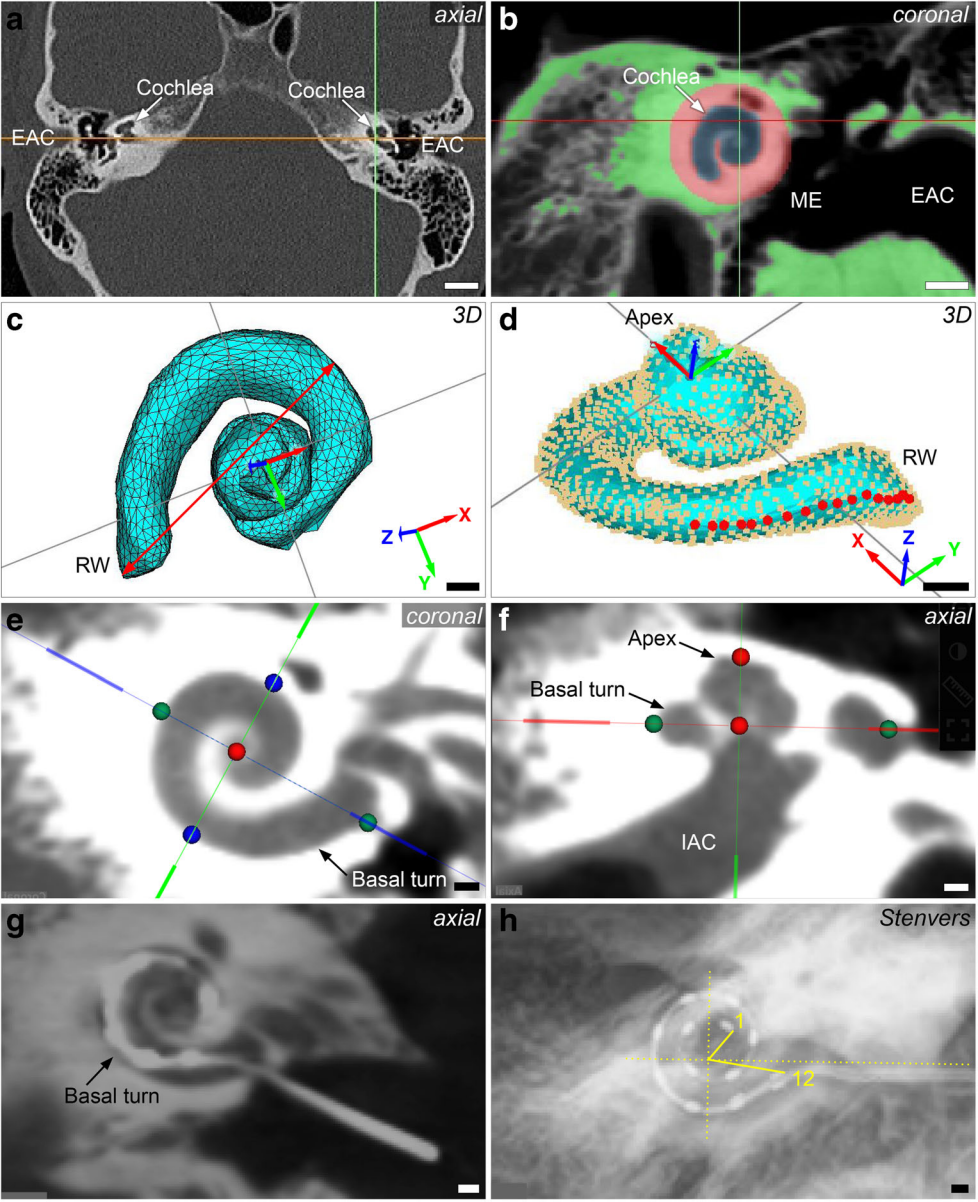
Cochlear duct length (CDL) measurement approaches. **a** Axial view of a high-resolution computed tomography (HRCT) scan in Materialise Mimics software. **b** Segmentation of a cone beam computed tomography (CBCT) scan in Materialise Mimics, threshold mask (green), region A (blue, cochlear fluid), and region B (red, otic capsule). **c** 3D model of the cochlea after segmentation. Red double arrow, measurement of the A-value from the round window (RW) to the opposite lateral wall (LW). Alignment: z-axis (blue), x-axis (red), y-axis (green) with z-axis pointing through the modiolus and the basal turn positioned in the xy-level. **d** Spline curve reconstruction from cloud point surface of the 3D model. Red dots: selected points on the LW. Alignment: z-axis (blue), x-axis (red) y-axis (green) with z-axis pointing through the modiolus and the basal turn positioned in the xy-level. **e **CBCT scan with view on the cochlea in the oblique coronal view in the otosurgical planning software, with measurements of the A-value (cochlear diameter; green dots) and the B-value (cochlear width; blue dots). **f** CBCT scan, with view on the cochlea in the axial view in the otosurgical planning software, red dots: determination of the height of the cochlea. **g** Postinsertion CBCT scan with view on the cochlea in the oblique coronal view in the otosurgical planning software showing the fully inserted electrode in the basal turn. **h** Postinsertion radiograph (Stenvers projection) showing the measurement of the insertion angle determined between the first (1.) and the twelfth (12.) electrodes. Yellow lines, angle of the first/twelfth electrode. EAC, external ear canal; IAC, internal auditory canal; ME, middle ear; RW, round window. Scale bars: 10 mm (**a**, **b**), 1 mm (**c**–**h**)

Based on these imaging data, cochlear parameters were measured using three different software-based methods: (1) length estimation based on a highly detailed 3D reconstruction of the cochlea using special 3D software (Materialise Mimics, version 21.0, Materialise NV), (2) length estimation based on the established A-value method, and (3) length estimation based on a recently introduced clinically approved otosurgical planning software based on a combination of A-value and B-value methods (Otoplan, version 3.0, Cascination) (Fig. 1).

### 3D segmentation

3D segmentation was performed using threshold analysis and a 3D model of the cochlea was reconstructed with Materialise Mimics Innovation Suite (version 21.0, Materialise NV). Analysis was based on the density of the fluid within the membranous labyrinth. The range was set to −1024 to +280 Hounsfield units (HU) for HRCT. The threshold ranges were set separately for each modality and had to be modified since tissue density in CBCT is usually specified in gray scale, not in HU [27]. The round window was marked as the starting point for the spline curve measurements. The cochlea was segmented manually in each slice by selecting the cochlear fluid spaces (“region A”), and the adjoining bony region, the otic capsule (“region B”) (Fig. 1B). A 3D model was created semi-automatically by the software. The lateral cochlear wall (LW) and A-value were measured using the Computer Simulation Technology Software (CST studio suite, Simulia, Dassault Systèmes) (Fig. 1C). A spline curve of the LW was created manually from the software-based point cloud model (Fig. 1D). To enable a full view onto the basal turn of the cochlea for later A-value determination, the 3D model was projected into a Cartesian coordinate system with the z-axis representing the modiolus (Fig. 1 C,D).

### A-value method

Escudé et al developed a formula to calculate the CDL based on the basal turn as pictured in HRCT images and the insertion angle (IA) θ of the CI electrode [11].

The A-value describes the distance between the round window and the opposite LW passing through the modiolus. To estimate the full CDL, θ is set to 900° imitating a CC of 100% and representing two and a half turns of the cochlea. The CDL is calculated as follows:

1 $$ \mathrm{LW}=2.62\times \mathrm{A}\times {\log}_{\mathrm{e}}\left(1+\frac{\uptheta}{235}\right) $$

2 $$ \mathrm{LW}=2.62\times \mathrm{A}\times 1.57 $$

### Otosurgical planning software-based method

Otoplan (Cascination AG) was used as otosurgical planning software. The software requires the user to provide defined anatomical landmarks capturing the diameter (A-value) and width (B-value: cochlear width perpendicular to the line segment of the A-value, intersection point modiolus) of the cochlea basal turn in the oblique coronal view which is obtained by rotating the axial, coronal, and sagittal axis until the basal turn is fully captured. A third parameter (H-value) defines the height of the cochlea between the apex and the base of the cochlea through the modiolus perpendicular to the A and B lines. The CDL calculation is based on the elliptic circular approximation and percentage of basal turn length (pBTL) as reported by Schurzig et al. [37].

3 $$ {\mathrm{CDL}}_{\mathrm{LW}}=\mathrm{pBTL}\left(\uptheta \right)\times \left[1.18\times \left(\mathrm{A}-0.7\right)+2.69\times \left(\mathrm{B}-0.7\right)-\sqrt{0.72\times \left(\mathrm{A}-0.7\right)\times \left(\mathrm{B}-0.7\right)}\right] $$

The software automatically estimates the CDL along the organ of Corti using a multiplication factor of 0.9 [27].

As an additional approximation to CDL values from higher imaging resolutions, a further estimation of the CDL was calculated mathematically by multiplying the CDL values by $$ \raisebox{1ex}{$10$}\!\left/ \!\raisebox{-1ex}{$9$}\right. $$ (CDL_10/9_) based on the findings of Schurzig et al who found CDL values approximately 10% larger, when segmented from high-resolution μCT images [23].

Additionally, the predicted CC and the predicted IA from the different imaging modalities were calculated as follows:

4 $$ \mathrm{CC}\left(\%\right)=\frac{31.5\mathrm{mm}}{\mathrm{CDL}\left(\mathrm{mm}\right)}\times 100\%. $$

5 $$ \mathrm{IA}\left({}^{\circ}\right)=\frac{\mathrm{CC}\left(\%\right)\times {900}^{\circ }}{100\%} $$

and compared to the reference.

All temporal bones were implanted with a CI electrode (MED-EL Flex soft electrode array, 31.5 mm, MED-EL GmbH) by a single surgeon (NMW). The electrode array was inserted using a posterior tympanotomy and round window approach.

Full insertion and correct scalar position were proven by postinsertion CBCT in all cases (Fig. 1G). The insertion depth and intracochlear position were verified by postoperative radiological imaging (Stenvers projection, Fig. 1H). The IA was measured using the method of Xu et al [5].

As a reference, the CDL was estimated based on the CI electrode insertion angle measured by radiographs in Stenvers projection (CDL_reference_) according to the following formula presuming a cochlear angle of 900°:

6 $$ \mathrm{CDL}\kern0.5em \left(\mathrm{mm}\right)=\frac{31.5\mathrm{mm}}{\mathrm{IA}\left({}^{\circ}\right)}\times {900}^{\circ }. $$

All measurements were performed by two independent examiners. One investigator (Ear, Nose and Throat [ENT] resident with 1 year of experience interpreting temporal bone imaging) performed all measurements after an instruction and training period under the supervision of two senior physicians (radiology consultant and ENT consultant, each with more than 6 years of experience). The second investigator was chosen according to the grade of experience in the use of each individual method (3D segmentation and A-value method: engineer with more than 5 years’ experience in the use of Materialise Mimics and 3D reconstructions from medical imaging; otosurgical planning software-based method and insertion angle determination: scientist specialized in otologic research with expertise of more than 4 years in the interpretation of temporal bone imaging). Only the measurements performed by the same person (ENT resident) were used for further analyses. Both investigators were blinded to the previous measurement. Consistency of the measurements was tested by determining the inter-rater reliability. 

### Statistical analysis

All statistical tests were selected before data collection. Statistical analyses were performed using Microsoft Excel (version 15.29, Microsoft Corporation) and Prism (version 8, GraphPad software). The significance level was set to *p* < 0.05. The assumption of normality was tested graphically using quantile-quantile plots. Data are presented as mean with standard deviation (SD) as well as absolute numbers with percentages. To compare means of the CDL between the measurement methods, a one-way analysis of variance (ANOVA) and Dunnett’s test were used to correct for multiple comparisons. To compare the CDL estimations obtained from the different measurement methods and CDL_reference_, Pearson’s correlation coefficient (PCC) was calculated. Furthermore, PCC was calculated between the CDL_SW_ and the A-value, B-value, and H-value. The agreement between the raters was determined by calculating the intra-class correlation coefficient (ICC).

## Results

A total number of 20 temporal bones was investigated. The means and standard deviations of the CDL depending on the different imaging modalities and measurement techniques as well as the calculated CC and the calculated IA from the different imaging modalities compared to the reference are shown in Table 1. A full insertion of a 31.5-mm electrode was achieved in all cases. No scalar dislocation and no tip fold-overs were observed. A mean CC of 74% was identified (SD 7.4%). The mean IA was 663° (SD 65.5°).

**Table 1:** Means and standard deviations of the different measurement techniques for (i) the estimated cochlear duct length (CDL), (ii) the calculated cochlear coverage (CC), and (iii) the calculated insertion angle (IA) compared to the reference

	3D segmentation		A-value method		Otosurgical planning software		Reference
	HRCT	CBCT	HRCT	CBCT	HRCT	CBCT	Stenvers projection
Mean CDL (mm)	35.5	37.0	36.0	38.2	37.0	37.6	43.2
SD CDL (mm)	1.4	1.3	1.2	2.0	1.8	1.7	4.3
Mean CC (%)	89.0	85.2	87.7	82.7	85.3	83.9	74
SD CC (%)	3.6	3.0	2.8	4.2	4.2	3.8	7.4
Mean AI (°)	800.8	766.6	789.4	744.5	767.8	755.5	663.3
SD IA (°)	32.6	26.9	25.3	37.9	37.3	34.1	65.4

**Fig. 2:**
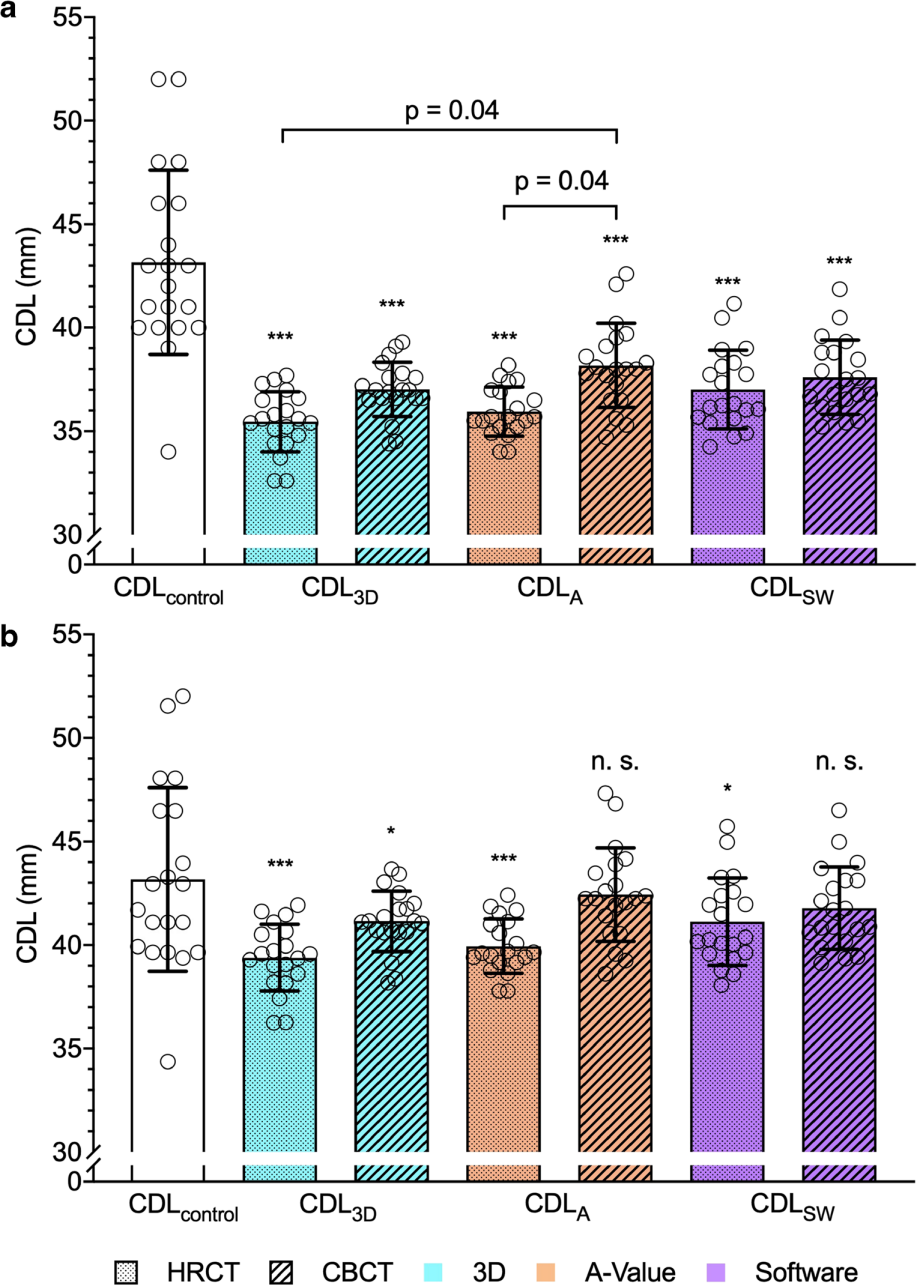
Scatterplot showing the distribution of values of CDL estimation of the individual techniques and the CDL_reference_. **a** Significant differences between the reference and every other method were found. Furthermore, significant differences between the A-value method obtained from CBCT and HRCT as well as between the A-value method obtained from CBCT and the 3D segmentation-based method obtained from HRCT were found. **b** Scatterplot after omitting correction factor. All measurement values approach to those from the reference. Asterisks mark *p* value of differences of the individual approaches compared to the reference. ***p < 0.001; *p < 0.05; n.s., not significant. Boxes indicate mean values. Whiskers indicate standard deviation

One-way ANOVA revealed differences between the individual CDL estimation methods (*F* (6, 133) = 25.24, *p* < 0.001). Post hoc analysis showed significant differences between the CDL_reference_, the CDL estimation based on 3D reconstruction from HRCT (CDL_3D-HRCT_; mean difference 7.7 mm, 95%CI 5.8–9.6 mm, *p* < 0.001), and from CBCT (CDL_3D-CBCT_; mean difference 6.1 mm, 95%CI 4.2–8.0 mm, *p* < 0.001), the CDL estimation based on an otosurgical planning software from HRCT (CDL_SW-HRCT_; mean difference 6.1 mm, 95%CI 4.2–8.1 mm, *p* < 0.001), and from CBCT (CDL_SW-CBCT_; mean difference 5.5 mm, 95%CI 3.6–7.5 mm, *p* < 0.001), the CDL estimation based on the A-value method from HRCT (CDL_A-HRCT_; mean difference 7.2 mm, 95%CI 5.3–9.1 mm, *p* < 0.001), and the from CBCT (CDL_A-CBCT_; mean difference 5.0 mm, 95%CI 3.1–6.9 mm, *p* < 0.001). Furthermore, differences between the CDL_A-HRCT_ and the CDL_A-CBCT_ (mean difference 2.2 mm, 95%CI 0.1–4.4 mm, *p* = 0.04) as well as between the CDL_3D-HRCT_ and the CDL_A-CBCT_ (mean difference 2.7 mm, 95%CI 0.6–4.9 mm, *p* = 0.04) were found (Fig. 2A).

When multiplying the individual values by $$ \raisebox{1ex}{$10$}\!\left/ \!\raisebox{-1ex}{$9$}\right. $$, differences between CDL_reference_ and CDL_A-CBCT_ as well as CDL_reference_ and CDL_SW-CBCT_ were no longer significant (Fig. 2B).

**Fig. 3:**
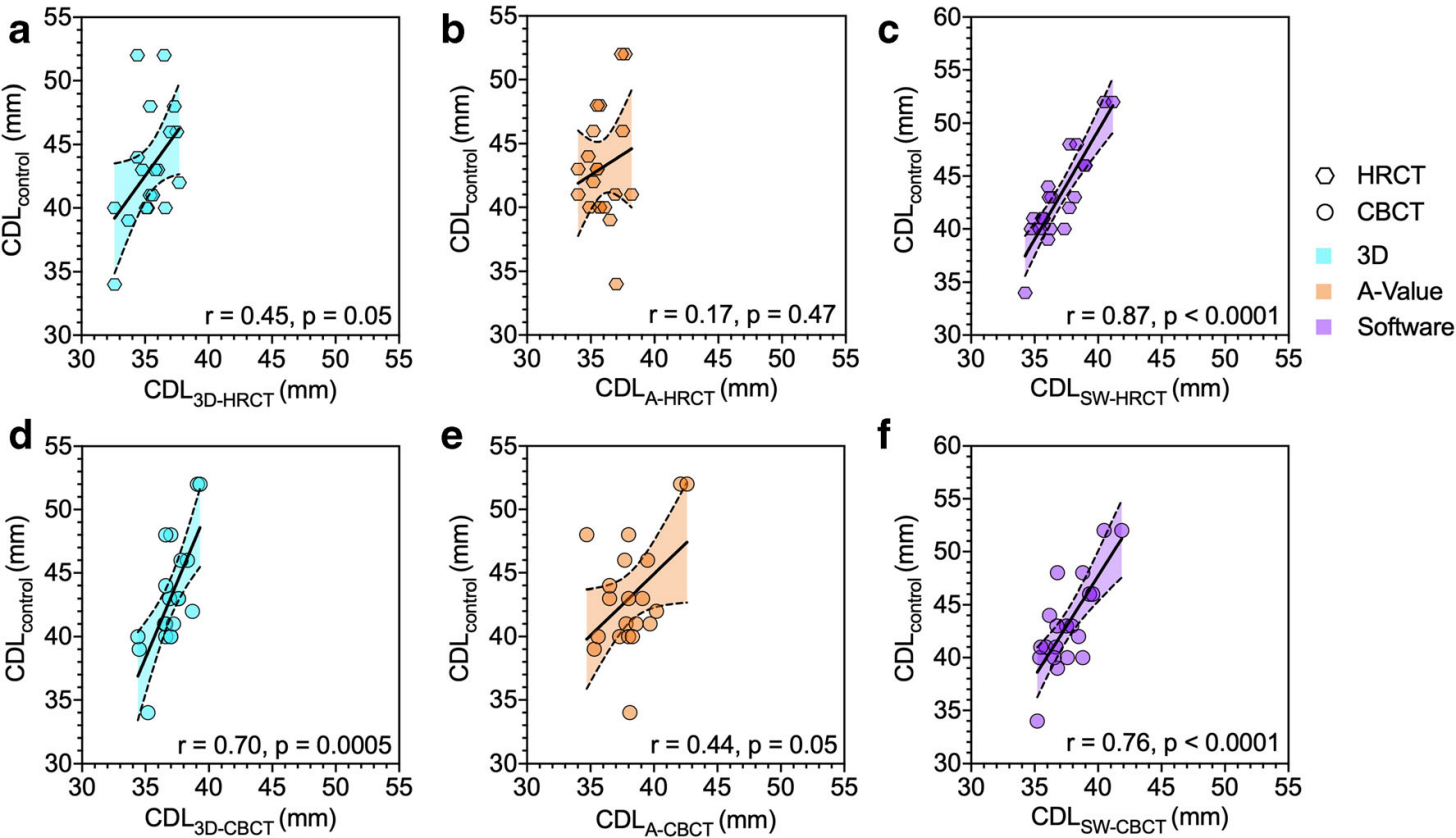
Correlations between the individual approaches and the reference. **a**–**c** Estimations obtained from HRCT using the 3D segmentation-based method (**a**), A-value-based method (**b**), and software-based method (**c**). **d**–**f** Estimations obtained from CBCT using the 3D segmentation-based method (**d**), A-value-based method (**e**), and software-based method (**f**). r, Pearson’s correlation coefficient. Line represents linear regression line. Dashed lines represent 95% prediction interval

The correlations between the CDL_reference_ and the different measurement techniques are shown in Fig. 3. These correlations were significantly different between the CDL_SW-HRCT_ and the CDL_3D-HRCT_ (*p* = 0.01) as well as between the CDL_SW-HRCT_ and the CDL_A-HRCT_ (*p* = 0.0007) and CDL_A-CBCT_ (*p* = 0.01). Furthermore, the correlations were significantly different between the CDL_3D-CBCT_ and the CDL_A-HRCT_ (*p* = 0.04) as well as between the CDL_SW-CBCT_ and the CDL_A-HRCT_ (*p* = 0.02).

Measured by HRCT and estimated by the otosurgical planning software, the PCC between the A-value and the CDL was 0.78 (*p* < 0.001), between the B-value and the CDL 0.95 (*p* < 0.001), and between the H-value and the CDL 0.70 (*p* = 0.0007). Measured by CBCT, PCC between the A-value and the CDL was 0.87 (*p* < 0.001), between the B-value and the CDL 0.97 (*p* < 0.001), and between the H-value and the CDL 0.80 (*p* < 0.001).

The inter-rater reliability among investigators was assessed using the ICC. The results are shown in Table 2. A fair inter-rater reliability for the CDL_3D-CBCT_, a good inter-rater reliability for the CDL_3D-HRCT_, a poor inter-rater reliability for the CDL_A-HRCT_, and an excellent inter-rater reliability per [29] for CDL_A-CBCT_, for CDL_SW-HRCT_, for CDL_SW-CBCT_, and for CDL_reference_ were found.

**Table 2:** Inter-rater reliability for the different measurement techniques. *ICC*, intra-class correlation coefficient; *ICC*_*A*_, intra-class correlation coefficient A-value; *ICC*_*B*_, intra-class correlation coefficient B-value

	CDL_3D-HRCT_	CDL_3D-CBCT_	CDL_A-HRCT_	CDL_A-CBCT_	CDL_SW-HRCT_	CDL_SW-CBCT_	CDL_reference_
ICC	0.71	0.43	0.29	0.87	0.94	0.94	0.87
ICC_A_	-	-	0.28	0.87	0.86	0.89	-
ICC_B_	-	-	-	-	0.93	0.87	-

## Discussion

The aim of this study was to investigate the accuracy and reliability of CDL measurements applied in clinical routine imaging. 3D segmentation was (i) intuitive and feasible even for less experienced users and (ii) the anatomical orientation is supported by the possibility to rotate the cochlea. This enables a full view onto the basal turn of the cochlea to apply the A-value method. 3D reconstruction complements conventional tomography and provides information about anatomical particularities that may influence the electrode choice [14, 28]. These information have usually been gained by histological examinations post mortem [30]. However, in the present study, the correlations of CDL_3D_ to the CDL_reference_ were only moderate and the inter-rater reliability varied between fair to good depending on the imaging modality. Inter-rater differences may be explained by the alignment of the cochlea in the coordinate system. Further disadvantages are its time effort and the need for reconstruction of cochlear fluid spaces. The HU threshold ranges need to be set manually, which may lead to inaccuracies especially when considering that CBCT and HRCT are not primarily used to assess soft tissue and fluids. Summarizing these observations, the method is considered prone to errors and has a flat learning curve.

The A-value method exhibited the poorest correlation with the CDL_reference_. Even though the method showed a high inter-rater reliability, only the basal turn is taken into account which leaves unattended the variability in the cochlear shape [7] and has a poorer correlation with the CDL compared to the B-value. This is in line with another experimental study demonstrating a significant underestimation of CDL from the A-value method compared to synchrotron imaging [31].

The otosurgical planning software-based method was considered intuitive and is the only presented method that is authorized for clinical use. Clinical standard imaging data may be used for the analysis. The required reformation to an oblique coronal view may be a barrier to obtaining accurate basal turn parameters as also remarked by Guenette [32]. Smaller differences in the imaging quality and the basal turn orientation may disproportionately influence final measurements. However, the method showed the highest correlations to the reference values and the best inter-rater reliability. This is in line with the findings of Canfarotta et al who reported an excellent agreement in the determination of the CDL [33]. The software uses a correction factor of 0.9 that is calculated to reduce the CDL from the LW size to the estimated size of the organ of Corti [27]. This factor is higher than the one suggested by Kawano et al [34] but may still lead to an underestimation of the CDL. Schurzig et al found CDL values approximately 10% larger, when segmented from high-resolution μCT images. It was concluded that the points placed onto the cochlear LW within CBCT images are located too close to the modiolus, resulting in a shorter estimated CDL [23]. This supports the findings of the current study where an underestimation of the CDL was observed. However, when applying an addition of 10%, the values approached the CDL_reference_ (Fig. 2B).

With a CDL range between 35.5 and 43.1 mm, the overall CDL range is in accordance to the values reported in the literature [24, 35]. However, only few studies considered the influence of imaging quality and resolution by applying the methods in different imaging modalities and lack a comparison of methods that are not authorized for clinical application to a clinically approved otosurgical planning software [22, 36]. Summarizing these results, a gold standard for the determination of the CDL is still missing.

This study showed the best correlations between CDL_SW-HRCT_ and CDL_reference_. Furthermore, this method is supported by an excellent inter-rater reliability. Overall CBCT measurements showed higher correlations to the CDL_reference_. This may be explained by the CBCT protocol used with a higher resolution and lower slice thickness than HRCT. Furthermore, the value of CBCT in CDL estimation is supported by Schurzig et al who found high correlation rates between CDL estimated by CBCT and μCT. Identifying the LW was easier in the basal region, but becomes more difficult in the middle and apical turn [37]. These findings highlight the importance of image resolution and may explain weaker correlations between the CDL and the cochlear height compared to the A-value and B-value. In this study, the width of the cochlea (B-value) had a larger influence on the CDL than the A-value. This is in line with another study describing a stronger correlation between the CDL and the B-value (*r* = 0.96) compared to the A-value (*r* = 0.63) [38]. A recent study by Oh et al showed correlations between the A-value and the IA comparable to this study but higher correlations between the A-value and the postoperative IA compared to the B-value [39]. These results may be explained by the lower IA with a mean of only 440° [32, 39]. Consequently, the determination of the basal turn length may be useful and leads to more accurate IA predictions when a shorter electrode is chosen. However, the impact of cochlear height and the radius of middle and apical turn may increase with a deeper electrode insertion or anatomical variations.

From the results of this study, the otosurgical planning software-based method is recommended for determining CDL in a normal shaped cochlea. However, this method only takes into account the A-value and B-value to determine the CDL. The H-value is not respected by the formula, even though a high correlation between H-value and CDL_SW_ was shown in this study. Regarding these findings, the accuracy of the software-based CDL measurement is likely to depend on a normal shaped basal turn and may consequently limit the use of the otosurgical planning software for the electrode choice in cases of inner ear malformation (IEM).

Concerning the imaging modality, we found the best agreement between the CDL_reference_ and CDL values obtained from HRCT. However, in clinical application, the decision whether to use CBCT or HRCT may additionally depend on other factors such as the patient age and radiohygiene. The CBCT has an optimum in combination of spatial resolution and radiation exposure for skull base examinations in the high-contrast range. However, CBCT is associated with a 2 to 3 times higher eye dose during temporal bone examinations [40]. Since the correlations between the CDL_reference_ and the CDL_SW-HRCT_ and between the CDL_reference_ and the CDL_SW-CBCT_ were not significantly different, the modality should be chosen in consensus with the neuroradiologist within the center.

This study is limited by a comparatively small number of temporal bones. Reproducibility of the results should be evaluated in larger prospective clinical trials. However, a number of 20 temporal bones is higher compared to other experimental studies using human temporal bone specimens [23, 41]. Varying estimated CDL may result from poor imaging resolution, insufficient referencing, and inadequate mathematical cochlea modelling. Small variances in the electrode position after implantation (LW versus perimodiolar) may lead to different values and estimation errors. Yet, CDL_reference_ obtained from Stenvers projection was considered to be the closest approximation to the clinical application of CDL estimations since it predicts the electrode position. Furthermore, MED-EL Flex soft electrodes are the thickest available electrodes and consequently are considered to exhibit the fewest variation inside the cochlear duct.

## Conclusion

Different values for the CDL depending on the imaging modality and the imaging processing software were observed. These variations may result from inaccuracies of the applied approaches and the mathematical CDL modelling. Further explanations are measurement errors or insufficient imaging resolution. Overall, the CDL was underestimated by each of the applied methods. As a consequence, all measurements would presumably have led to an electrode choice of rather too short electrodes. Particularly, in cases of electric acoustic stimulation where a precise electrode choice according to the residual hearing is desirable, these findings are of importance. Concerning treatment decisions based on CDL measurements, a method considering both A-value and B-value that is approved for clinical use has to be recommended for patients with a normal cochlea shape. Furthermore, we provide evidence that any correction factor may be omitted. All present models for CDL estimation appear not to achieve the level of precision needed for an appropriate estimation of pitch match or CC in residual hearing by electrode selection.

## Abbreviations

ANOVA: One-way analysis of variance
CBCT: Cone beam computed tomography
CC: Cochlear coverage
CDL: Cochlear duct length
CDL_3D_: Cochlear duct length 3D reconstruction
CDL_A_: Cochlear duct length A-value method
CDL_CBCT_: CDL estimated from CBCT
CDL_HRCT_: CDL estimated from HRCT
CDL_reference_: Cochlear duct length reference
CDL_SW_: Cochlear duct length software-based method
CI: Cochlear implant
FOV: Field of view
HRCT: High-resolution computed tomography
HU: Hounsfield units
IA: Insertion angle
ICC: Intra-class correlation coefficient
IEM: Inner ear malformation
LW: Lateral cochlear wall
pBTL: Percentage of basal turn length

## Declarations

### Guarantor

The scientific guarantor of this publication is Nora M. Weiss.

### Statistics and biometry

No complex statistical methods were necessary for this paper.

### Methodology

• Experimental

• Performed at one institution
